# Plant-based proteins for infant formula: findings and recommendations from the ILSI Europe workshop

**DOI:** 10.3389/fnut.2025.1677243

**Published:** 2025-11-19

**Authors:** Kasper A. Hettinga, Chris H. P. van den Akker, Nils Billecke, Renée Cardinaals, Ching-Yu Chang, Didier Dupont, Matthew Furber, Kate Grimshaw, Paula Hallam, Peter de Jong, Julia K. Keppler, Carolien A. van Loo-Bouwman, Cath Mersh, Kelly A. Mulder, Elena Oliveros, Susan Ozanne, Iben Lykke Petersen, Daniel Tomé, Yvan Vandenplas, Fred van de Velde, Sascha C. A. T. Verbruggen

**Affiliations:** 1 Food Quality & Design Group, Wageningen University & Research, Wageningen, Netherlands; 2 Department of Pediatrics-Neonatology, Amsterdam UMC - Emma Children's Hospital, Amsterdam, Netherlands; 3 Cargill R&D Centre Europe BV, Vilvoorde, Belgium; 4 Earth Systems and Global Change Group, Wageningen University & Research, Wageningen, Netherlands; 5 International Life Science Institute European Branch, Brussels, Belgium; 6 INRAE l'Institut Agro, STLO, Rennes, France; 7 Danone Research & Innovation, Utrecht, Netherlands; 8 Manchester Metropolitan University, Manchester, United Kingdom; 9 Northern Care Alliance NHS Foundation Trust, Manchester, United Kingdom; 10 Tiny Tots Nutrition, London, United Kingdom; 11 Van Hall Larenstein University of Applied Sciences, Leeuwarden, Netherlands; 12 NIZO Food Research B.V., Ede, Netherlands; 13 Laboratory of Food Process Engineering, Wageningen University & Research, Wageningen, Netherlands; 14 Yili Innovation Center Europe, Wageningen, Netherlands; 15 Cath Mersh Communications, Aarhus, Denmark; 16 Abbott, Granada, Spain; 17 Department of Clinical Biochemistry, Cambridge University, Cambridge, United Kingdom; 18 Department of Food Science, University of Copenhagen, Copenhagen, Denmark; 19 AgroParisTech, Université Paris-Saclay, Paris, France; 20 KidZ Health Castle, UZ Brussel, Brussels, Belgium; 21 Department of Neonatal and Pediatric Intensive Care, Erasmus MC Sophia Children's Hospital, Rotterdam, Netherlands

**Keywords:** plant-based proteins, infant formula, pediatric nutrition, nutritional adequacy, sustainability, food regulation, protein quality

## Abstract

This Review Article summarizes outcomes from the ILSI Europe expert workshop on plant-based proteins in infant formula, held in November 2024. Experts from academia, clinical nutrition, and food science evaluated the current use and future potential of plant-based protein sources in infant formula, considering nutritional adequacy, allergenicity, sustainability, processing technologies, and regulatory constraints. While soy and hydrolyzed rice proteins are already approved and in use, emerging sources such as pea, lentil, and faba beans show promise but require further validation of their amino acid profiles, digestibility, safety, and suitability for infants. Key research priorities identified include the development of improved protein extraction methods, *in vitro* digestion and allergy modeling, and targeted clinical studies. This review synthesizes current evidence and expert perspectives to support the development of sustainable, nutritionally adequate plant-based infant formulas.

## 1 Introduction

Human milk is the gold standard source of infant nutrition. Yet, for those infants in the world who are only partially breastfed or not breastfed at all, there is an unquestionable need for the best possible alternative. Over generations, commercial infant formula, primarily based on cow's milk, has become widely perceived as the most suitable alternative to meet this need.

In recent years, a new movement toward less animal-derived food has developed. Concerns about the environment, climate change, animal welfare and human health have sparked a growing demand for plant-based food and beverages (1). Pan-European market research has found that sales of plant-based drinks are increasing steadily (2).

Such concerns may persuade some parents to buy plant-based formulas for their children. However, questions remain about the ability of plant-based proteins to meet infants' nutritional requirements. In November 2024, International Life Sciences Institute (ILSI) Europe hosted a workshop to explore the implications of a shift from cow's milk protein to plant-based protein in formula for infants below the age of 12 months. The first 4 to 6 months of an infant's life are particularly critical. At this stage, the infant's gut is immature, which means protein sources must be easy to digest.

The workshop's primary objectives were to consider the environmental sustainability of plant-based alternatives to dairy proteins and identify gaps in the knowledge about their nutritional quality, health implications, processing, and feasibility in formula for infants below 12 months. Discussions covered the plant-based protein already approved for commercial hypoallergenic formula (3) and potential new protein sources.

Production of an entirely vegan formula is complex, as several ingredients in addition to cow's milk protein must be replaced. To simplify discussions, the scope of the workshop was limited to the proteins in infant formula alone.

The workshop participants included scientists, nutritionists, clinical practitioners and R&D specialists from academia, clinical practice and industry (Table 1).

**Table 1:** Workshop participant list.

**S. No**.	**Name**	**Affiliations**
1	Kasper Hettinga	Wageningen University & Research
2	Carolien van Loo	Yili Innovation
3	Didier Dupont	INRAE
4	Sascha Verbruggen	Erasmus MC Sophia Children's Hospital
5	Susan Ozanne	University of Cambridge
6	Elena Oliveros	Abbott
7	Kelly Mulder	Danone Research & Innovation
8	Nils Billecke	Cargill R&D Centre Europe BV
9	Iben Lykke Petersen	University of Copenhagen
10	Keppler Julia	Wageningen University & Research
11	Fred van de Velde	NIZO
12	Daniel Tomé	Paris-Saclay University
13	Yvan Vandenplas	UZ Brussel
14	Chris van den Akker	Amsterdam UMC
15	Kate Grimshaw	Manchester Metropolitan University/Northern Care Alliance NHS Foundation Trust
16	Paula Hallam	Tiny Tots Nutrition
17	Peter de Jong	Van Hall Larenstein University of Applied Sciences/NIZO
18	Renée Cardinaals	Wageningen University & Research
19	Matthew Furber	Danone Research & Innovation
20	Euridice Castaneda Gutierrez	H&H
21	Janna van Diepen	Reckitt Benckiser Mead Johnson
22	Marieke Abrahamse	Danone Research & Innovation
23	Marco Turini	dsm-firmenich
24	Annalisa Segat	Kerry
25	Eduardo Romero Ramírez	Kerry
26	Benjamin Voiry	Roquette
27	Catherine Lefranc-Millot	Roquette
28	Ching-Yu Chang	ILSI Europe
29	Maria Tonti	ILSI Europe
30	Cath Mersh	Scientific writer

## 2 Plant-based infant formula—the state of play and research questions

Current conventions and regulations are a relevant starting point when discussing the possibilities to substitute the cow's milk protein in infant formula with proteins from a plant source. At present, soy protein isolate and hydrolyzed rice protein isolate are the sole plant proteins approved for use in infant formulas within the European Union (EU) (4–6).

Across the Atlantic, the US Food & Drug Administration only recognizes soy as an appropriate plant-based component in formulas for healthy, full-term infants (7). In the UK, however, the official position is that soy-based infant formulas should not be the first choice for infants with cow's milk-related sensitivities and disorders due to perceived health risks (8).

Food Standards Australia New Zealand has set another example. In 2021, the authority approved a new plant-based infant formula that contains pea protein and intact rice protein (9). The commercial product is marketed as a soy-free choice for parents.

Soy protein has been used in infant nutrition for more than a century and, over this time, modern soy-based formulas have been developed to meet all standards for infant safety and nutrition (10). Based on current knowledge, the European Society for Pediatric Gastroenterology Hepatology and Nutrition (ESPGHAN) Committee on Nutrition has concluded that soy is a suitable protein source in infant formula. The committee does recognize, however, soy's inferiority to cow's milk due to lower digestibility and bioavailability and its unsuitability for infants with food allergy below the age of 6 months (5).

Once infants reach 6 months of age, dietary intake becomes more diverse as infants no longer rely solely on human milk or formula. This explains a less restrictive approach to the composition of plant-based toddler formula, where protein sources of interest currently include buckwheat, almond, oat and chickpea (11).

**Figure 1:**
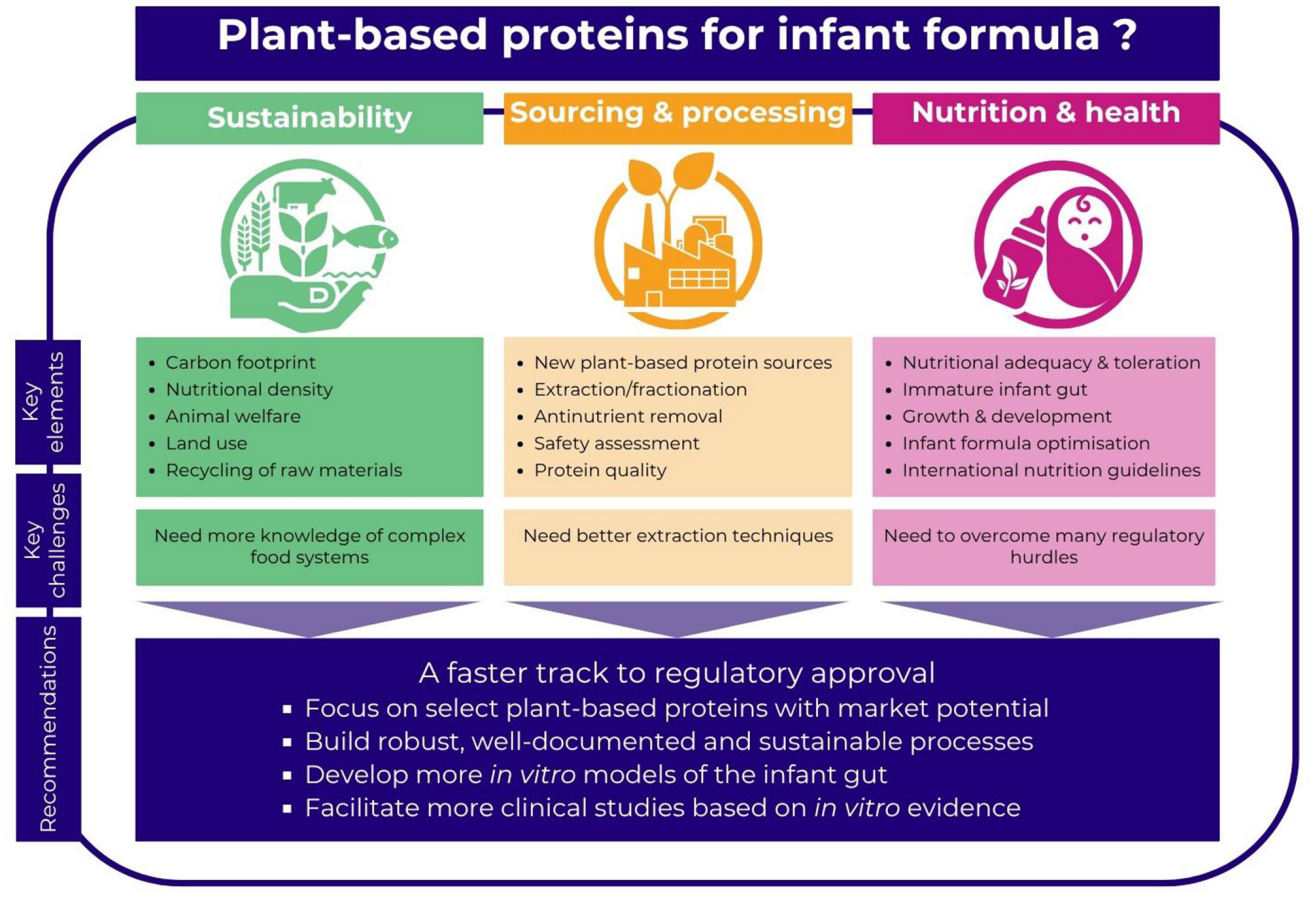
Plant protein alternatives for infant formula: key considerations and recommendations.

In response to the plant-based food trend, emerging sources of plant-based protein are the subject of considerable research (12). One of the questions stemming from this research is which, if any, of these emerging proteins are safe and suitable in formula products for infants up to 6 or 12 months of age—and, from that, which are likely candidates for future regulatory approval. The ILSI Europe workshop considered these new possibilities and related limiting factors from three perspectives: sustainability, sourcing & processing, and nutrition & health (Figure 1).

**The research questions**

In relation to these three perspectives, the workshop explored the following research questions:

- What do we know about the potential environmental impact of a shift toward plant-based proteins in infant formula?
- What are the challenges when processing plant-based protein sources and how does processing impact safety and nutritional quality?
- Can plant-based proteins provide adequate, well-tolerated nutrition that supports infant growth and development in the short and long-term?

## 3 The sustainability perspective: what do we know about the potential environmental impact of a shift toward plant-based proteins in infant formula?

The adoption of the Paris Agreement at the UN Climate Change Conference in 2015 definitively put climate change on the global agenda. In the years that have followed, many companies—including leading infant formula manufacturers—have joined the Science-Based Targets Initiative, which provides a pathway to achieving the goals of the Paris Agreement by reducing greenhouse gas emissions (13).

At first sight, the environmental rationale for choosing plant-based proteins over dairy proteins seems clear. A simple calculation shows that dairy protein contributes around 13% of the total carbon footprint of cow's milk infant formula (14). When cow's milk production is compared with the cultivation of plant-based protein crops, the greenhouse gas emissions of cow's milk are notably higher per hectare (15). Yet, this land-use evaluation is an incomplete picture.

### 3.1 From LCA to n-LCA

The traditional life cycle assessments (LCAs) behind such figures only give insight into the environmental impact of the switch from dairy to plant-based protein. They do not consider other aspects, such as contribution to nutrient intake. This shortcoming was recognized by the Food and Agriculture Organization of the United Nations (FAO), which led a project to identify new approaches to capturing sustainability and health impacts within a complex food system and, in that way, enable reliable comparisons of food products (16). The project's detailed report sets out best practices for integrated environmental and nutritional LCAs (n-LCAs). Briefly explained, these require that food products are compared on the basis of their primary function—nutrient provision—rather than on their weight. This approach has shown that the carbon footprint of semi-skimmed milk is, in fact, lower than that of most unfortified plant-based beverages due to its higher nutrient density. The footprint of fortified plant-based beverages, however, is lower than that of semi-skimmed milk (17). Although no plant-based infant formulas were included in this study, the findings are relevant as plant-based formulas must be fortified to meet strict nutritional requirements, benefiting their n-LCA value.

### 3.2 Factors in a circular system

Another study has gone beyond LCAs, developing a model for a radical transformation of food systems. Called the Circular Food Systems (CiFoS) model, this applies circularity principles to producing sufficient food while safeguarding human and planetary health. The model highlights a number of additional factors of importance when comparing the sustainability of dairy and plant-based foods. Use of marginal grasslands, sustainable manure management, and reuse of food waste and food production by-products can reduce the environmental footprint of a food system where animals have a role (18, 19).

### 3.3 Sustainability perspective: summary

To summarize, the true picture of plant-based protein's sustainability is likely to differ greatly from any simple environmental calculation. For now, in the context of our current food system, the extent to which plant-based proteins have a sustainability advantage is hard to predict.

## 4 The sourcing and processing perspective: what are the challenges when processing plant-based proteins and how does processing impact safety and nutritional quality?

The sourcing and processing of plant-based proteins has a strong bearing on their sustainability and nutritional quality in general and their safe use in infant formula in particular. Over time, numerous plant-based sources have emerged as candidates for replacing or complementing animal-based proteins in the diet. Each one requires attention to determine its nutritional content, technical functionality and level of consumer acceptance—not to mention its availability for commercial production.

Within the EU, the sustainability agenda focuses on reducing dependence on imported protein crops such as soy and boosting European plant protein production (20). Starch-rich legumes all have potential, along with oil crops. Proteins from microalgae, precision fermentation, and others lie further ahead on the horizon (21).

Considerable research along with long and costly approval processes are necessary before any of these new protein sources can be used in infant nutrition (22). Against this backdrop, it makes sense to focus on a limited number of promising candidates.

### 4.1 Pure proteins by extraction

Infant formula production entails a series of steps that ensure the purity and nutritional composition of each component. Concerning the protein content, fractionation is necessary to obtain high-quality protein fractions from the raw material.

Wet extraction technologies, including isoelectric precipitation and ultrafiltration, can be applied to steer protein purity, yield and techno-functionality in terms of heat stability, solubility, and emulsification. Ultrafiltration is considered the milder of these processes, producing a protein of higher purity (23). When legumes are subjected to a wet fractionation process, starch, fiber, and proteins are the major fractions obtained. Common unit operations are dehulling, milling, and alkaline extraction of the proteins. An additional defatting step using solvents may be included to improve functionality and purity, although pulses are relatively low in fat. The alkaline protein extract still contains soluble carbohydrates and fibers and is further purified by ultrafiltration or through isoelectric precipitation of the proteins (24). Depending on the process parameters, wet fractionation can produce both protein concentrates and isolates. Purity after wet fractionation can be 80% or higher.

Dry fractionation, on the other hand, enriches the protein phase, retaining some residual starch and fibers. This produces an ingredient with a more moderate protein content of between 35% and 65% (25). Because no water is used, dry fractionation *per se* is considered to have the lowest environmental impact (23), though the lower protein content can suggest otherwise when using an n-LCA approach, as more ingredient is required to achieve the same protein output.

Pea (*Pisum sativum*), lentil (*Lens culinaris*), and faba bean (*Vicia faba*) are increasingly investigated as sustainable, plant-based protein sources due to their availability in Europe, relatively high protein content (typically 20%−30%, with faba bean reaching up to 35%), and favorable digestibility. Moreover, these pulses are capable of nitrogen fixation, thereby reducing the need for nitrogen fertilizer (24).

### 4.2 The antinutrient challenge

A challenge common to all plant proteins is the varied presence of antinutritional factors (ANFs), which impact protein digestibility and utilization on ingestion and the bioavailability of other essential nutrients. For this reason, processing strategies are required to remove and/or deactivate ANFs and, thereby, improve the nutritional quality (26). Dehulling is an important initial step in this process, as it physically removes the ANFs present in legume hulls and cereal bran, such as tannins and phytate. Wet fractionation has also proven more effective than dry fractionation in this regard (27, 28). This is partly because of the soaking step, which reduces the water-soluble ANFs.

Other approaches to ANF reduction in plant proteins have been tested, revealing a series of pros and cons. Heat treatment reduces phytate and denatures protease inhibitors (29), germination reduces phytic acid through endogenous enzyme activation (30), and fermentation can reduce ANFs while enhancing nutritional quality (31, 32). All these processes have a significant impact on protein techno-functionality, both positive or negative.

Successful processing of plant proteins is about hitting the right balance. Studies have shown that ANFs are not always a disadvantage as the negative impact on nutrient absorption may be offset by other biologically positive outcomes (33). For example, human milk itself is known to contain protease inhibitors (34, 35). One study, which has investigated the possibility that ANFs are not necessarily a hindrance, used lentil protein in a model infant formula. Despite the presence of trypsin inhibitors in the lentil protein isolate, the lentil-based formula had a similar digestibility and functionality to commercial soy and rice-based formulas. Lentil protein does not, however, provide sufficient cysteine, methionine and tryptophan to satisfy minimum regulatory requirements for the amino acid content of infant formula. For this reason, the model infant formula was supplemented with these amino acids (36).

While pea and lentil protein isolates contain common ANFs, faba bean additionally contains two pyrimidine glycosides: vicine and convicine. These compounds are of particular concern as they may trigger favism in individuals with glucose-6-phosphate dehydrogenase deficiency. Their concentrations can be significantly reduced through processing techniques such as soaking, germination, and thermal treatment (28). Furthermore, plant breeding efforts have successfully developed low-vicine and low-convicine cultivars, making faba bean protein increasingly suitable for food and nutrition applications.

### 4.3 The safety question

In every discussion of plant protein processing, the question of safety should be kept in mind. From a spoilage and safety perspective, the use of plant-based proteins should always be accompanied by strict procedures for risk assessment and mitigation.

As new-born infants are vulnerable consumers, the safety of all types of infant formula is the primary concern. Similarly, it is crucial that infant formula products satisfy all the nutritional requirements of infants. This means that, after processing, high-quality plant proteins care those that contain adequate, bioavailable amounts of all nine indispensable amino acids.

### 4.4 Measurement of protein quality

Two methods are used to measure protein quality: Protein Digestibility Corrected Amino Acid Score (PDCAAS), which is based on fecal digestibility, and Digestible Indispensable Amino Acid Score (DIAAS), based on ileal digestibility. DIAAS is the most precise but difficult to measure in infants (37).

At present, the indispensable amino acid pattern of human milk is recognized as the international standard for the protein quality of infant formula (38). To determine the protein quality of follow-on formula for children from the age of 1 year, the UN Food & Agriculture Organization (FAO) recommends the PDCAAS method (39). Further research is needed to assess plant protein digestibility in infants and the bioavailability of essential amino acids in plant-based formula. This should include longitudinal clinical studies monitoring growth, neurodevelopment, and gut health, as well as *in vitro* digestion models simulating infant gastrointestinal conditions of protein digestion and peptide and amino acid release.

### 4.5 Sourcing and processing perspective: summary

Strict regulations on infant formula composition and the long road to regulatory approval provide a strong rationale for seeking out the novel plant-based proteins with the biggest commercial potential. These promising candidates should then be the primary focus of scientific investigation.

Many research gaps remain to be filled. The limitations of existing fractionation technology highlight some of the challenges related to extracting proteins from plant raw materials while maintaining their nutritional integrity. Simply put, extraction techniques must be further developed to maximize the purity of the protein while minimizing processing-induced damage.

## 5 The health and nutrition perspective: can plant-based proteins provide adequate, well-tolerated nutrition that supports infant growth and development in the short and long-term?

The need for digestible proteins to support healthy growth and development is at its highest during infancy. A joint WHO/FAO/UNU Expert Consultation has defined daily protein and amino acid requirements in detail (40), with human milk as the gold standard for infants. Codex Alimentarius and the European Food Safety Authority have made use of similar definitions in their respective standard and opinion on the essential composition of infant formula (41, 42).

Infant formula contains more protein than human milk to ensure infant amino acid requirements are met (43). The required protein content of soy-based infant formula is slightly higher than cow's milk infant formula as plant proteins are generally lower in essential amino acids and DIAAS values than dairy proteins (44).

However, it may be possible to combine multiple plant sources to balance out individual shortfalls in specific amino acids. In one theoretical study, an optimized blend of six plant proteins—potato, sisymbrium sp., corn gluten meal, pea albumin, Brazil nut and napin—was found to be a 97.9% match with the reference profile for indispensable amino acids in human milk (45). Although none of these plant proteins are currently realistic options for application in commercial infant formula, the blend principle is a useful one that could be applied to other, more widely available plant protein sources with a complementary composition of essential amino acids. Such blends could be a means to reduce the total protein content of infant formula, bringing it closer to the protein level of human milk.

### 5.1 Cow's milk allergy and the potential for tolerance

Studies of optimized plant protein blends are of interest for the development of appropriate plant-based formulas both for healthy infants and for infants with cow's milk allergy (CMA), for whom conventional infant formula products are not suitable.

CMA typically appears during the first 6 months of life and has a noticeably higher prevalence among formula-fed than breastfed infants (46). In many parts of the world, the first-choice recommendation for managing CMA in non-exclusively breastfed infants is extensively hydrolysed formula (eHF) based on whey or casein (3, 47, 48).

Findings from a UK assessment show that 29% of formula-fed infants with suspected CMA continued to present symptoms on an eHF (49). Two plant-based solutions are currently approved for use—soy protein formula (SPF) and hydrolyzed rice protein formula (HRPF). In the widely accepted Diagnosis and Rationale for Action against Cow's Milk Allergy (DRACMA) and American Academy of Pediatrics (AAP) guidelines, HRPF and SPF appear as second-choice options for managing specific CMA manifestations (50).

GA^2^LEN and DRACMA guidelines generally advise against using SPF in infants under 6 months with CMA, due to potential allergenicity and limited clinical data The ESPGHAN Committee on Nutrition, however, does not specify a strict age limit, but advises against SPF as a first-line option in infants with food allergies, although soy is considered a safe second choice alternative for economic and palatability reasons (50). A comprehensive review of soy sensitization found no strong evidence of increased allergy risk in infants under 6 months (51). Alternative plant protein sources would offer parents additional choice, nonetheless. An increasingly used alternative to eHF, HRPF has been found to be both well tolerated and an efficient source of nutrition in five clinical studies (52–56). The growth of infants fed HRPF due to suspected CMA has also been seen to be comparable with that of healthy infants (57, 58). Overall, research to date has given no reason to question the safety of HRPF, though the limited number of clinical trials conducted with HRPF in healthy infants means the present findings may not be conclusive.

At present, no other plant proteins, including pea, lentil, and faba bean for example, are approved for use in infant formula for infants under 1 year, and there is a lack of data on their allergenicity in this age group. Before such proteins can be considered for commercialization, extensive evaluation of their allergenic potential—including *in vitro* assays, animal studies and, ultimately, infant clinical trials—will be essential. Moreover, conflicting data on soy allergenicity between regions, for example USA vs. Europe, highlight the need for such assessments to take possible geographical differences in sensitization and tolerance into account.

### 5.2 Investigations of hybrid formulas

Several *in vitro* studies have investigated the partial replacement of cow's milk protein with plant-based proteins in infant formula (59–61). Although processing challenges concerning infant formula functionality still need to be resolved, hybrid formulas do appear to have potential from a nutritional perspective (59). This has been shown using an *in vitro* model of infant digestion, which found that an infant formula with 50% cow's milk and 50% faba bean protein had a similar PDCAAS to a reference milk-based formula (60). A study by the same research group used degree of protein hydrolysis and amino acid bioaccessibility as indicators of protein digestibility. In this case, model infant formulas with 50% faba bean or pea protein had a similar digestibility to the reference milk-based formula, while the digestibility of model formulas with 50% rice or potato protein was significantly lower (61). *In vivo* studies have yet to confirm these results.

### 5.3 Health and nutrition perspective: summary

A number of internationally recognized institutions have specified the daily protein and amino acid requirements of infants. To satisfy these specifications, commercial plant-based infant formulas tend to have a slightly higher total protein content than cow's milk formula. Several amino acids may also be individually added.

Due to the varying amino acid content of individual plant-based proteins, one future strategy may be to combine them in optimized blends. In this way, plant-based formula could be tailored to complement the amino acid composition of cow's milk formula, eliminating the need to increase the total protein content. Obtaining regulatory approval for a blend, however, is likely to be more complicated than for a single protein source.

Another possibility is to combine dairy protein with plant-based proteins as a means of optimizing the amino acid composition of cow's milk infant formula, bringing it closer to that of human milk. But, again, many regulatory hurdles remain, requiring further *in vitro* and *in vivo* studies.

## 6 Conclusion and recommendations—where can we go from here?

Plant-based proteins as a category are attracting growing interest across the global food sector. The ILSI Europe workshop focused on their current use and future potential opportunities in infant formulas.

With regard to sustainability, LCA studies have evolved from being purely concerned with the environmental footprint of animal-based vs. plant-based products and now include nutrient density (17). At present, there is no evidence that plant-based proteins are nutritionally equivalent (5).

Although the precise sustainability advantage of replacing dairy proteins with plant-based proteins in infant nutrition has yet to be clarified, the metrics used to measure sustainability plus the prevailing food system are two key factors.

### 6.1 The rationale for continued research

Nevertheless, there are several good reasons for continuing to explore the possibilities. First and foremost among them is the need to meet the rising parental demand for safe, well-tolerated and nutritionally adequate plant-based infant formulas. From a business perspective, the gradual but continuous tightening of regulations on infant nutrition around the world is another important factor. The disappearance of soy-based infant formula from the UK market is one outcome of this.

From the perspective of protein accessibility, locally sourced plant-based proteins are relevant in regions where milk is in short supply or where there is a desire to become less dependent on soy imports (21). The unique quality of each plant protein source may provide the basis for optimized protein combinations with the required amino acid composition (45).

In most markets, the choice of approved plant-based infant formulas is currently largely limited to soy protein or hydrolysed rice protein (50). Australia is one of the few exceptions, having approved an infant formula based on sprouted pea and rice protein ingredients for infants with or without CMA (62). Other plant proteins with good nutritional and digestibility potential include faba, lentil and quinoa (8, 36, 63).

### 6.2 Considerable remaining hurdles

However, there are many hurdles to overcome before more novel plant proteins can be put forward for regulatory approval in commercial infant formula products. As discussed, existing processing methods for extracting proteins or removing ANFs may compromise the proteins' nutritional quality and techno-functionality (64). In addition, limited clinical data is available on the digestibility, absorption, and tolerance of plant-based proteins by infants, along with their ability to support normal growth and development in the short and long-term.

**Figure 2:**
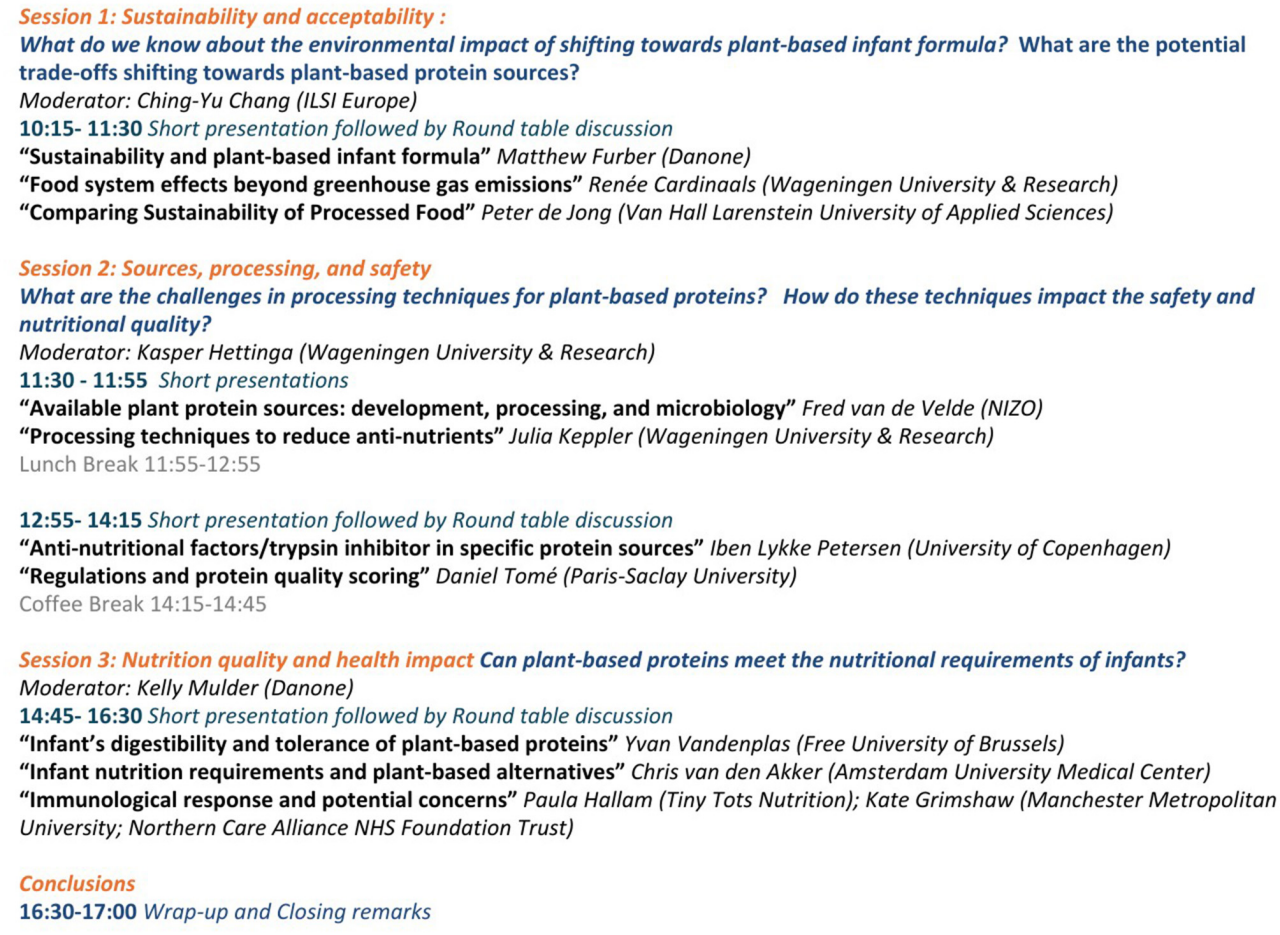
Workshop program.

In view of these challenges, the ILSI Europe workshop concluded by drawing up key recommendations for diversifying the range of plant-based protein sources available for infants (Figure 2).

### 6.3 Recommendations of the ILSI Europe workshop

- **Select a few promising candidates**
  Further research is necessary to identify novel plant-based proteins with the greatest potential for infant formula, including *in vivo* studies of nutritional quality. A sharp focus on a small number of the most promising candidates is essential to build the body of evidence necessary for regulatory approval. Additionally, business considerations, such as raw material availability and processing costs must be integrated in the selection process to ensure feasibility and sustainability.
- **Build robust and sustainable processes**
  To achieve commercial success, reliable and well-documented processes are required to produce plant-based proteins with uniform nutritional quality and the highest safety standards, ensuring they are well-tolerated by immature infant guts. The incorporation of environmental mitigation strategies in the design will ensure sustainability and minimize ecological impact.
- **Develop more, reliable** ***in vitro*** **models**
  Animals are commonly used in pre-clinical trials and, in many cases, serve as valuable infant gut models for measuring digestion (65–67). However, the ethical concerns surrounding animal research are growing. Therefore, it is crucial to develop additional, reliable *in vitro* models to measure plant-based protein digestibility and mimic absorption in the infant gut. These models will help reduce the reliance on animal testing and could provide more accurate data for human applications. The computer-controlled TNO gastrointestinal Model (TIM) is one existing example of these (68).
- **Facilitate more clinical studies**
  Recruitment of infants for clinical trials is prerequisite to progress. With the backing of strong *in vitro* evidence, it is hoped that more parents can be persuaded to enroll their infants in clinical trials that investigate plant-based protein digestibility and absorption. Clinical trials should also document that the growth and development pattern of infants fed a plant-based formula is at least equivalent to that of infants on cow's milk formula. Conclusive clinical documentation is indispensable to the regulatory approval process, ensuring the safety and efficacy of these plant-based proteins for infant nutrition.

## Author's note

This work was conducted by an expert group (EG) of the European branch of the International Life Sciences Institute, ILSI Europe. According to ILSI Europe policies, the EG is composed of at least 50% external non-industry members. The complete composition of the EG can be found on the ILSI Europe website (https://ilsi.eu/scientific-activities/nutrition/early-nutrition-and-long-termhealth/). Experts are not paid for the time spent on this work.
